# The Role of Tranexamic Acid Use in Reduction Mammaplasty: A Systematic Review and Meta-Analysis

**DOI:** 10.1093/asjof/ojaf035

**Published:** 2025-05-03

**Authors:** Zainah Abdulbari Alhebshi, Sham Radwan Sabbagh, Zainab Jasim AlQurain, Aya Omar Bamuqabel, Dana Jamal Dahlan, Rawan Abdulmuati Rajoub, Danah Osama Fallatah, Banan Abdulmuati Rajoub, Hanan Muhammed Ismail Wasaya, Ezdehar Fallatah

## Abstract

Reduction mammoplasty (RM) decreases breast size but may lead to complications such as hematoma. Local tranexamic acid (TXA) is applied during surgery to minimize bleeding during or after the procedure. This systematic review, conducted in August 2024 using PubMed, Ovid Medline, and Ovid Embase, assessed the impact of local TXA on RM. The Methodological Index for Nonrandomized Studies and the Revised Cochrane risk of bias (RoB2) tools were employed to assess the risk of bias. Data analysis was performed using RevMan software. In this meta-analysis of 5 studies involving 608 patients (1216 breasts) undergoing RM, 46.8% of breasts were treated with TXA. Local TXA significantly reduced the 24-h drain output (mean difference −11.49, 95% CI, −15.71, −7.26; *P* < .00001). Higher TXA concentrations (20 and 25 mg/mL) significantly reduced hematoma rates (odds ratio [OR] 0.09, 95% CI, 0.03, 0.30; *P* < .0001), whereas lower concentrations showed no similar effect. TXA also reduced the complication rates in RM (OR 0.63, 95% CI, 0.50, 0.80; *P* = .0002), although its impact on drain utilization, duration, and seroma rates was not statistically significant. Local TXA showed promising results, because it reduced the 24 h drain output, overall occurrence of postoperative complications, and when used in higher concentrations, decreased the hematoma formation. However, we need further randomized controlled trials to confirm the efficacy of different concentrations of TXA in RM.

**Level of Evidence:** 3 (Therapeutic)

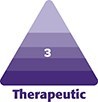

According to the Aesthetic Society Cosmetic Data Bank, 75,782 reduction mammoplasty (RM) procedures were performed in 2023, underscoring its safety and efficacy as the recommended therapeutic approach for managing breast hypertrophy.^1,2^ Although RM is generally a safe and effective procedure, postoperative complications may arise, including hematoma, seroma, infection, wound-healing issues, skin loss, painful scars, fat necrosis, nipple loss, and reoperations, particularly because of postoperative bleeding.^3,4^ Postoperative hematoma is a common complication of RM, with an incidence of 0.3% to 2%.^5^ This complication can significantly impact recovery and may necessitate additional interventions, highlighting the need for strategies to reduce its occurrence and improve patient outcomes.

One such strategy involves inhibiting fibrin clot dissolution by blocking the conversion of plasminogen to plasmin, thereby reducing fibrin degradation and subsequent bleeding. Tranexamic acid (TXA), an antifibrinolytic agent, exerts this effect.^6^ TXA is employed in various surgical fields through multiple administration routes, including intravenous (IV), topical, oral, and local infiltration.^7^ In a recent systematic review, Scarafoni showed that TXA effectively reduces blood loss and postoperative inflammation in aesthetic surgery without increasing thrombotic risk, thereby facilitating recovery regardless of the administration route.^8^ In addition, Om et al reported that the use of IV TXA is a safe and effective method for reducing hematoma rates in RM patients.^9^

However, a gap remains in the literature: no systematic reviews or meta-analyses have assessed the effect of locally administered TXA (topically applied or infiltrated) in RM. Therefore, we conducted a local TXA efficacy evaluation to give a comprehensive and relevant recommendation on its use in RM.

## METHODS

### Literature Search

The systematic review followed the Cochrane review protocols and the Preferred Reporting Items for Systematic Reviews and Meta-Analysis (PRISMA) guidelines.^10,11^ Additionally, it adhered to the International Prospective Register of Systematic Reviews statement.^12^ During August 2024, we conducted a detailed search in the electronic databases PubMed, Ovid Medline, and Ovid Embase with no time limits. The search algorithms used were: (topical tranexamic acid OR topical TXA OR TXA OR tranexamic acid) AND (reduction mammoplasty OR breast reduction OR mammoplasty OR reduction mammaplasties OR mammaplasties).

### Study Selection

The electronic search results were processed using the software program Rayyan (Rayyan Systems Inc., Qatar), which was employed to select and exclude articles.^13^ A team of 5 researchers independently screened the abstracts and selected potentially relevant studies. Any discrepancies led to the article being advanced to a full-text review. The criteria for inclusion in the systematic review were as follows: (1) studies published up until August 2024 without any time restrictions; (2) studies reporting the number of patients assessed; (3) studies published only in English; (4) studies comparing the use of local TXA in managing RM with a control group; (5) studies reporting outcomes relevant to the clinical question; and (6) original research articles, including randomized controlled trials (RCTs), cross-sectional studies, prospective and retrospective cohort studies, case–control studies, case reports, and case series. Studies were excluded if they did not meet the inclusion criteria.

### Screening and Data Extraction

Full-text studies were obtained and evaluated for adherence to the inclusion and exclusion criteria by 2 independent authors, whereas any unresolved disagreements regarding the articles were addressed by the senior author. The screening process, along with the reasons for exclusion, was meticulously documented using the PRISMA flowchart. Data from each included article were retrieved by 2 investigators, who screened the articles for our inclusion criteria and extracted the following information: (1) study details, including the last name of the first author, year of publication, country, study design, and follow-up period; (2) participant information such as the total number of participants (breasts), gender, age, number of TXA cases and controls (no-TXA), and TXA dosage; (3) operative and drain-related outcomes, including the necessity of postoperative drainage, total drain output, drain duration, and operative duration; and (4) postoperative complications, including hematoma formation, seroma formation, and overall complications. For every article we included, we assigned a level of evidence (LOE) based on the criteria set out by the American Society of Plastic Surgeons (ASPS) in their guidelines for rating LOE and grading recommendations.^14^

### Bias Assessment

Two reviewers independently assessed the risk of bias in all included studies. For observational studies, they used the Methodological Index for Nonrandomized Studies (MINORS), and for RCTs, they applied the Revised Cochrane risk of bias (RoB2) tool.^15,16^ To ensure accuracy, a third reviewer verified their assessments.

For the MINORS tool, we focused specifically on comparative studies. This involved examining 12 specific domains, with each one rated on a scale from 0 to 2, making the highest possible score 24. Using MINORS, scores were categorized as follows: 0 to 4 = very low quality; 5 to 8 = low quality; 9 to 12 = moderate quality; 13 to 16 = high quality; and 19 to 24 very high quality.^15^

On the other hand, the RoB2 tool evaluated 5 key areas that assessed different aspects of how the trial was designed, carried out, and reported. These areas were examined using “signaling questions,” which helped gather the necessary information to judge bias. Based on the responses, an algorithm categorized the studies into 3 risk levels: “low” (meaning low risk of bias across the board), “some concerns” (meaning there was a concern in at least 1 area), or “high” (meaning there was either a high risk in at least 1 area or concerns in several areas).

### Statistical Analysis

A single author with statistical expertise performed the meta-analysis using Review Manager (RevMan) Version 5.4 (Cochrane, London, England). The analysis involved calculating mean differences (MDs) or odds ratios (ORs), along with 95% CIs, with statistical significance determined by a *P*-value of <.05. Heterogeneity among the studies was evaluated using *χ*^2^ and *I*^2^ tests within the RevMan software. If significant heterogeneity was identified (*I*^2^ > 50%), a random-effects model was employed; otherwise, a fixed-effects model was applied when heterogeneity was minimal.

## RESULTS

### An Overview of the Reviewed Studies’ Characteristics

**Figure 1.**
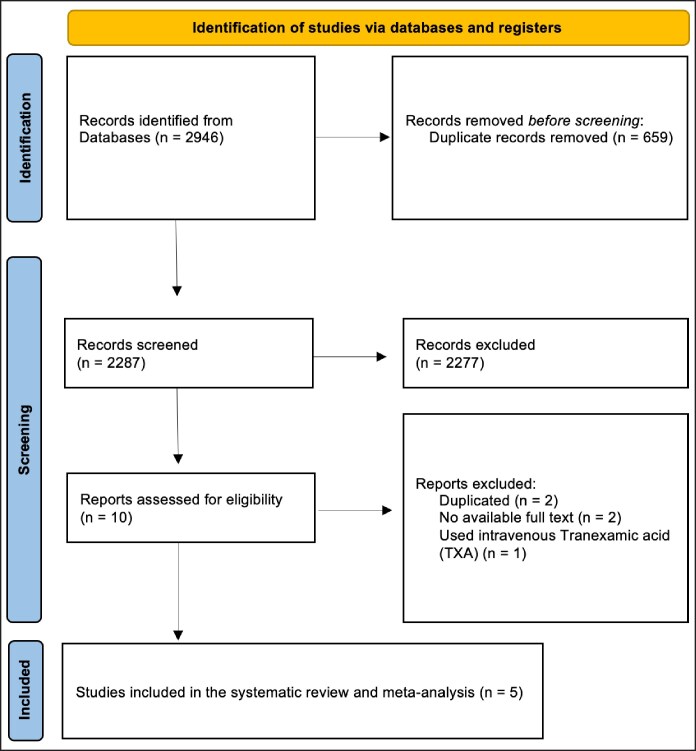
The Preferred Reporting Items for Systematic Reviews and Meta-Analysis (PRISMA) flowchart of the reviewed studies.

From an initial pool of 2946 records identified across primary databases (PubMed, Ovid Medline, and Ovid Embase), 10 studies underwent eligibility assessment. Among these, 5 studies (2 RCTs, 2 retrospective studies, and 1 prospective study) met our inclusion criteria (Figure 1, Table 1).^17-21^ These studies collectively involved 608 female patients and 1216 breasts undergoing RM. Of these, 569 breasts (46.8%) were treated with local TXA, whereas 647 breasts (53.2%) in the control group were treated with a placebo. The concentration of locally applied TXA varied among the studies, ranging from 3 to 25 mg/mL (Table 2). According to ASPS scaling, 2 studies were classified as LOE I, 2 as Level II, and 1 as Level III (Table 1). We evaluated several operative and drain-related outcomes (Table 3), including the use of drains, drain output after 24 h, drain duration, and operative duration. However, only one study reported the mean duration of RM, limiting the analysis of this outcome.^18^ Additionally, we assessed postoperative complications in both groups (Table 4).

**Table 1.** Overview of the Included Studies Characteristics

Article	Country	Study design	Total participants, *n* (breast)	Gender		Age (years), mean ± SD, (median) (range)	Follow-up period, *n*	ASPS scale for grading
				Male	Female			
Ausen et al^17^	Norway	RCT	28 (56)	0	28	(45) (18-67)	90 days	I
Nichols et al^18^	United States	Retrospective study	81 (162)	0	81	30 ± 13.44	18 months	III
Pathak et al^19^	India	Prospective study	25 (50)	0	25	43 (25-67)	NA	II
Sipos et al^20^	Finland	Retrospective study	376 (752)	0	376	NA	NA	III
Yao et al^21^	United States	RCT	98 (196)	0	98	34.14 ± 12.87	30 days	I

ASPS, American Society of Plastic Surgeons; NA, not available; RCT, randomized controlled trial; TXA, tranexamic acid.

**Table 2.** Overview of TXA Concentrations and Methods of Application in Included Studies

Article	TXA concentration (mg/mL)	Method of application
Ausen et al^17^	25	TXA (20 mL) was topically applied to the wound surface before closure
Nichols et al^18^	3	TXA was infiltrated into each breast as part of the tumescent solution (200-300 mL)
Pathak et al^19^	25	TXA (20 mL) was topically applied to 1 breast before closure, whereas the other breast received a saline placebo
Sipos et al^20^	20	TXA was topically applied through irrigation of the wound surface (25 mL per breast) before closure
Yao et al^21^	10	TXA (20 mL) was topically applied to the raw wound surface using an Angiocath catheter after hemostasis and before closure

TXA, tranexamic acid.

**Table 3.** Overview of Operative and Drain-Related Data in Reduction Mammoplasty: Comparison Between Groups With and Without Application of TXA

Article	Number of participants		Drain utilization, *n*		Drain output after the first 24 h (mL), mean ± SD		Operative duration (min), mean ± SD		Drain duration (days), mean	
	TXA cases, *n* (breast)	Controls, *n* (breast)	TXA cases, *n* (breast)	Controls, *n* (breast)	TXA cases, *n* (breast)	Controls, *n* (breast)	TXA cases, *n* (breast)	Controls, *n* (breast)	TXA cases, *n* (breast)	Controls, *n* (breast)
Ausen et al^17^	28 (28)	28 (28)	27 (27)	25 (25)	15.05 ± 9.66	27.89 ± 27.43	NA	NA	NA	NA
Nichols et al^18^	41 (82)	40 (80)	NA	NA	NA	NA	162	157	8 ± 4.48	6 ± 4.94
Pathak et al^19^	25 (25)	25 (25)	25 (25)	25 (25)	13.6 ± 4.87	24.84 ± 10.65	NA	NA	NA	NA
Sipos et al^20^	168 (336)	208 (416)	13 (26)	59 (118)	NA	NA	NA	NA	NA	NA
Yao et al^21^	98 (98)	98 (98)	98 (98)	98 (98)	NA	NA	NA	NA	6.7 ± 1.477	6.9 ± 1.477

NA, not available; *n*, number of patients; TXA, tranexamic acid.

**Table 4.** Overview of the Postoperative Complications in Reduction Mammoplasty: Comparison Between Groups With and Without Application of TXA

Article	No. of participants		Hematoma, *n*		Seroma, *n*		Presence of complications, *n*	
	TXA cases, *n* (breast)	Controls, *n* (breast)	TXA cases, *n* (breast)	Controls, *n* (breast)	TXA cases, *n* (breast)	Controls, *n* (breast)	TXA cases, *n* (breast)	Controls, *n* (breast)
Ausen et al^17^	28 (28)	28 (28)	1 (1)	9 (9)	1 (1)	1 (1)	13 (13)	21 (21)
Nichols et al^18^	41 (82)	40 (80)	2 (4)	3 (6)	1 (2)	2 (4)	15 (30)	17 (34)
Pathak et al^19^	25 (25)	25 (25)	0	0	0	0	0	0
Sipos et al^20^	168 (336)	208 (416)	1 (2)	12 (24)	3 (6)	5 (10)	88 (176)	132 (264)
Yao et al^21^	98 (98)	98 (98)	2 (2)	1 (1)	0 (0)	0 (0)	26 (26)	35 (35)

*n*, number of patients; TXA, tranexamic acid.

### Use of Drains

**Figure 2.**
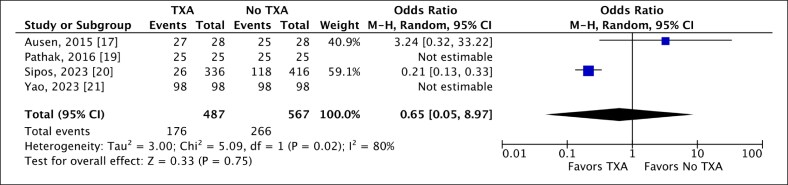
A random-effect forest plot of 95% CI showing a statistically insignificant association between the use of local tranexamic acid (TXA) and the drain utilization in reduction mammoplasty.

Four studies compared intraoperative drain placement in RM between TXA and control groups.^17,19-21^ Drains producing >0 mL output defined the drain-utilization group, whereas those with no drains or 0 mL output were grouped as functionally equivalent. Among 487 breasts in the TXA group, only 36.14% used drains vs 46.91% of 567 controls. Our analysis did not reveal a statistically significant association between the local application of TXA and a reduction in drain utilization in RM (OR 0.65, 95% CI, 0.05, 8.97; *P* = .75). The high heterogeneity observed suggested inconsistency among the studies (Figure 2).

### Drain Output 24 h Postoperatively

**Figure 3.**
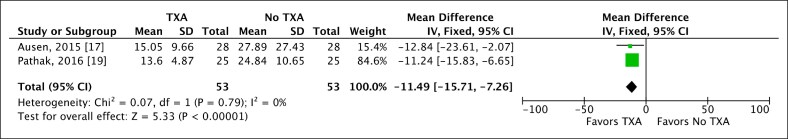
A fixed-effect forest plot of 95% CI showing a statistically significant association between the use of local tranexamic acid (TXA) and the reduced drain output in milliliters when measured 24 h postoperatively in reduction mammoplasty.

Two studies measured the total drain output in milliliters following RM, with and without the application of local TXA.^17,19^ The mean total drain output was 14.366 ± 7.74 mL in the TXA group and 26.45 ± 21.10 mL in the control group. Our analysis demonstrated that the application of local TXA significantly reduced the drain output observed 24 h after RM (MD −11.49, 95% CI, −15.71, −7.26; *P* < .00001; Figure 3). The low heterogeneity observed suggested consistency between the studies.

### Drain Duration

**Figure 4.**
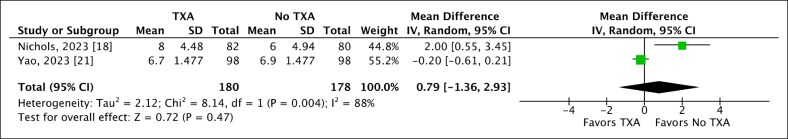
A random-effect forest plot of 95% CI showing a statistically nonsignificant association between the use of local tranexamic acid (TXA) and the drain duration following reduction mammoplasty.

Two studies reported the mean duration of drain placement following RM, comparing the TXA and non-TXA groups.^18,21^ The mean duration until drain removal was 7.29 ± 3.27 days for the TXA group and 6.50 ± 3.5 days for the control group. Our analysis showed no statistically significant association between the application of local TXA and the duration of drain placement (MD 0.79, 95% CI, −1.36, 2.93; *P* = .47). The high heterogeneity observed suggested inconsistency among the studies (Figure 4).

### Hematoma Formation

**Figure 5.**
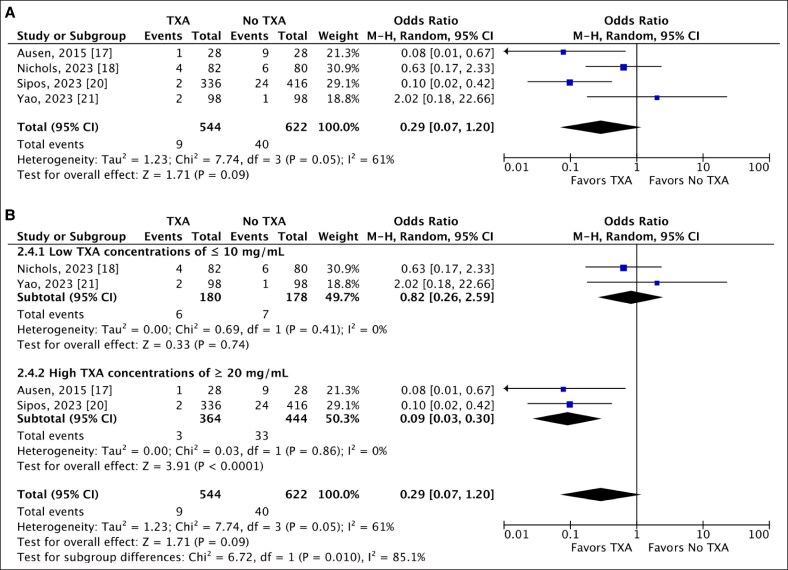
(A) A random-effect forest plot of 95% CI showing a statistically nonsignificant association between the use of local tranexamic acid (TXA) and the occurrence of hematoma following reduction mammoplasty (RM). (B) A subgroup analysis based on the different concentrations of TXA, showing a statistically significant reduction in hematoma occurrence following RM when TXA is used in higher concentrations.

Four included studies reported the incidence of hematoma following RM, comparing the local TXA group to the control group.^17,18,20,21^ Hematoma occurred in 1.65% of breasts in the TXA group, compared with 6.43% in the control group. However, this association did not reach statistical significance. Our analysis yielded an OR of 0.29 (95% CI, 0.07-1.20) and a *P*-value of .09. High heterogeneity was noted, indicating inconsistency among the studies (Figure 5). To identify the source of heterogeneity, a subgroup analysis based on the concentration was performed. Two articles used a relatively lower TXA concentration (3 and 10 mg/mL), whereas the remaining 2 articles used a higher concentration of (20 and 25 mg/mL).^17,18,20,21^ Notably, both analyses demonstrated low heterogeneity (*I*^2^ = 0%). Subgroup analysis of higher TXA concentrations revealed a statistically significant reduction in hematoma occurrence following RM, with an OR of 0.09 (95% CI, 0.03-0.30; *P* < .0001). Conversely, lower TXA concentrations were not significantly associated with hematoma occurrence (OR 0.82, 95% CI, 0.26-2.59; *P* = .74; Figure 5).

### Seroma Formation

**Figure 6.**
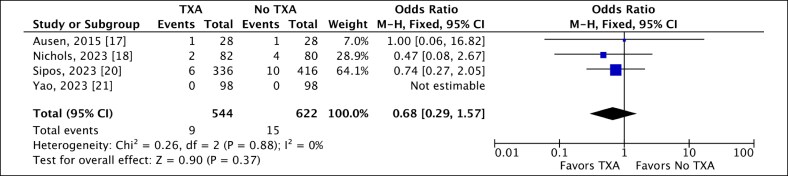
A fixed-effect forest plot of 95% CI showing a statistically nonsignificant association between the use of local tranexamic acid (TXA) and the occurrence of seroma following reduction mammoplasty.

Similarly, 4 studies reported seroma rates following RM, comparing local TXA with the control group.^17,18,20,21^ Seroma occurred in 1.65% of cases in the TXA group, compared with 2.41% in the control group. Our analysis showed no statistically significant association between the application of local TXA and the occurrence of seroma (OR 0.68, 95% CI, 0.29, 1.57; *P* = .37). The low heterogeneity observed suggested consistency among the results (Figure 6).

### Presence of Complications

**Figure 7.**
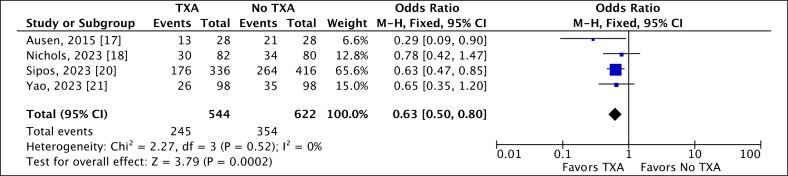
A fixed-effect forest plot of 95% CI showing a statistically significant association between the use of local tranexamic acid (TXA) and the reduced occurrence of complications following reduction mammoplasty.

Four included studies reported the number of complications in each group following RM.^17,18,20,21^ Complications included hematoma, seroma, wound infections, cellulitis, fat necrosis, asymmetry, wound dehiscence, and other minor skin issues. Interestingly, 45% of breasts in the TXA group experienced postoperative complications, compared with 56.9% in the control group. Our analysis indicated a statistically significant association between the application of local TXA and a reduced complication rate following RM (OR 0.63, 95% CI, 0.50-0.80; *P* = .0002). The low heterogeneity observed suggested consistency among the results (Figure 7).

### Risk of Bias Assessment

The Cochrane Risk of Bias Assessment Tool for Randomized Trials (RoB2) was used to evaluate RCTs.^17,21^ The included RCTs were considered to have a low risk of bias (Table 5). On the other hand, nonrandomized studies were assessed using the MINORS tool.^18-20^ We used the 12 fixed domains for comparative studies, with a maximum score of 24. The total scores of the studies were 21, 22, and 19, respectively, with an average score of 20.67, indicating high study quality.

**Table 5.** Review Authors’ Judgments About Each Risk of Bias Item for Each Included Randomized Controlled Trials Studies According to the Revised Cochrane Risk of Bias (RoB2) Assessment Tool

Article	Bias arising from the randomization process	Bias because of deviation from intended intervention	Bias because of missing outcome data	Bias in measurement of the outcome	Bias in selection of the reported result	Overall RoB
Ausen et al^17^	Low	Low	Low	Low	Low	Low
Yao et al^21^	Low	Low	Low	Low	Low	Low

The items with the lowest scores were the appropriateness of the follow-up period for the study's aim (score of 0 or 1), the unbiased assessment of the study endpoint (score of 0 or 2), and the baseline equivalence of groups (score of 1 or 2). The remaining items received the highest scores (2 in all studies; Table 6).

**Table 6.** Review Authors’ Judgments About Each Risk of Bias Item for Each Included Comparative Nonrandomized Study According to the Methodological Index for Nonrandomized Studies Assessment Tool

Article	Nichols et al^18^	Pathak et al^19^	Sipos et al^20^
A clearly stated aim	2	2	2
Inclusion of consecutive patients	2	2	2
Prospective collection of data	2	2	2
Endpoints appropriate to the aim of the study	2	2	2
Unbiased assessment of the study endpoint	0	2	0
Follow-up period appropriate to the aim of the study	1	0	0
Loss to follow-up <5%	2	2	2
Prospective calculation of the study size	2	2	2
An adequate control group	2	2	2
Contemporary groups	2	2	2
Baseline equivalence of groups	2	2	1
Adequate statistical analyses	2	2	2
Total score	21	22	19

Scores: 0 = not reported, 1 = reported but inadequate, and 2 = reported and adequate.

## DISCUSSION

RM is a relatively crucial, frequent, and safe procedure to manage macromastia. However, common complications following this procedure certainly exist, such as wound-healing problems, hematoma or postoperative bleeding, seroma, and infections.^3,4,22^ TXA, a synthetic lysine analog, has been frequently used for hemostasis in various surgical fields.^7,23^ TXA inhibits the activation of plasminogen by blocking the lysine-binding sites of plasminogen, leading to increased clot stabilization and thus reducing blood loss.^24^ Thus, TXA's unique properties make it an incredibly valuable adjunct in plastic surgery, specifically RM.^8^ In this systematic review, we focused on the local application of TXA in RM for several reasons. First, the IV administration of TXA is associated with an elevated risk of complications, such as thromboembolic events, particularly in patients with predisposing conditions. For example, Foster et al reported that intraoperative IV TXA use in sarcoma patients increased the risk of pulmonary embolism and deep vein thrombosis.^25^ Additionally, Colomina et al recommend topical administration of TXA as a viable alternative to the IV route, especially in patients with uncertain thrombotic risk.^26^ This method minimized systemic absorption, thereby reducing the risk of complications. Second, there is a notable lack of systematic reviews and meta-analyses that specifically address the local application (topical or infiltrated) of TXA.

Our analysis showed that using local TXA did not significantly reduce the need for drain utilization following RM (*P* = .75). Although the ASPS clinical practice guideline recommends against the routine use of drains in breast reduction surgeries, this recommendation relies on 3 RCTs that consistently show no improvement in postoperative complication rates with drains. However, these studies have notable methodological weaknesses, including inadequate reporting of treatment effect sizes, lack of consideration for critical outcomes like major hematomas, and variations in drain removal protocols, which limit their generalizability.^27^ According to Phillips et al, most plastic surgeons—93% out of 4669—use volume criteria to decide when to remove drains, with >86% doing so when the drain output is <30 mL per 24 h in breast reconstructive surgeries.^28^ Ausen et al, however, used a threshold of <40 mL per 24 h for drain removal and found that TXA can reduce drain fluid production after RM to levels below this cutoff for almost all patients.^17^ This finding suggested that TXA may eliminate the need for drains in RM, although further evidence is needed to confirm its efficacy. Avoiding drains is particularly beneficial because drainage systems can cause complications such as drain migration, blockage from clotted blood, discomfort, and pain at the drain site, which can lead to longer hospital stays and increased costs.^29^ Additionally, drain sites can leave scars and may act as potential sources of infection.^30^

We found a significant reduction in 24 h postoperative drain output with the application of local TXA (*P* < .00001). In an RCT, Safran et al reported a 30.5% reduction in mean drain output for mastectomy patients undergoing direct-to-implant breast reconstruction when TXA was used.^31^ On the other hand, in a systematic review, Huynh et al concluded that TXA, regardless of the administration route in breast surgery, did not affect drain output.^32^ Our analysis showed no statistically significant difference in drain duration between the TXA and control groups (MD 0.79 days, *P* = .47), with slightly longer drain retention in the TXA group. This contrasts with Safran et al's findings, which reported a 1.4 day difference in drain removal times between cases and controls.^31^ Similarly, Weissler et al found that applying topical TXA in implant-based breast reconstruction significantly reduced the duration of drain use compared with the control group.^33^

One of the most serious bleeding issues following breast surgery is postoperative hematoma, which may necessitate a trip back to the hospital or operating room for drainage or evacuation.^34^ It has been demonstrated that TXA reduces the risk of hematoma following breast surgery.^35,36^ Our meta-analysis indicated that local TXA may serve as an effective hemostatic agent in reducing hematoma formation (1.65% vs 6.43%), although not statistically significant. A similar result was observed by Weissler et al in breast reconstruction.^33^ Interestingly, when analyzing outcomes based on TXA concentrations, we observed that higher concentrations (≥20 mg/mL) significantly reduced hematoma occurrence (*P* < .0001), whereas lower concentrations did not yield the same effect. Therefore, further RCTs focusing on TXA concentrations are warranted. Another potential complication following RM is seroma formation. Because seromas are a frequent complication in breast surgery overall, the precise incidence following breast reduction is not well-defined, although it is estimated to fall between 1% and 3%.^33,37^ Although studies in implant-based reconstruction and breast cancer surgery suggested TXA may reduce seroma risk, our analysis in RM found only a nonsignificant numerical trend (1.65% TXA vs 2.41% control; OR 0.68, *P* = .37).^33,38^ Preclinical evidence posits that TXA stabilizes fibrin clots to seal lymphatic leaks and modulates cytokines like IL-6 and TNF-α, but these mechanisms may be insufficient in RM, where seroma pathogenesis involves prolonged inflammation and lymphatic disruption beyond TXA's transient antifibrinolytic window.^39^ Further research should explore optimized TXA dosing, sustained delivery, or adjunct therapies targeting later-phase seroma drivers.

Moreover, the overall complication rate was 45% in the TXA group and 57% in the control group, demonstrating an absolute reduction of 11.9% in complications with TXA use (*P* = .002). These complications included hematoma, seroma, wound infections, cellulitis, fat necrosis, asymmetry, wound dehiscence, and other minor skin issues.^17,18,20,21^ A systematic review and meta-analysis reported major complication rates ranging from 2.4% to 14% and minor complications from 2.4% to 69%.^40^ This discrepancy may be attributed to differences in study inclusion criteria, patient demographics, surgical techniques, and definitions of complications.

The elevated complication rates observed in our study could impact the generalizability of our findings. It suggests that our study population might have had a higher baseline risk, which could influence the observed efficacy of TXA in reducing complications. Therefore, although our results indicate a potential benefit of TXA, they should be interpreted with caution, considering the higher overall complication rates. Despite these limitations, our findings align with previous evidence suggesting that TXA can reduce postoperative wound complications. For instance, Oertli et al stated that TXA may be used to reduce the frequency of postoperative wound complications following surgery for breast cancer.^38^

Although concerns existed regarding TXA thrombotic risks, none were observed in our analysis, although caution was recommended for patients with recent myocardial infarction or coronary interventions. A correlation between TXA and seizures was theorized by its ability to cross the blood–brain barrier and antagonize the inhibitory gamma-aminobutyric acid receptor type A (GABA_A_) in the brain, with dose-dependent incidence reported in cardiac surgery.^41-43^ However, no seizures occurred in our analysis, potentially reflecting differences in TXA dosing or surgical context. Topical TXA, which minimizes systemic exposure, may further mitigate these risks compared with IV administration, warranting exploration in RM.^41,44^

The strengths of this review included stringent inclusion criteria, adherence to PRISMA guidelines, and the inclusion of 2 RCTs. Although the results offer valuable insights into treatment, several limitations should be acknowledged. First, the variability in study design and data reporting introduces heterogeneity among the included studies, which could impact the comparability of results. Some studies provided insufficient details on certain aspects. Second, the review encompasses only 5 studies, potentially limiting the reliability of the findings. Third, 2 studies were retrospective, which introduces potential biases and confounding factors that may affect the interpretation of the results. Fourth, the lack of long-term follow-up data in many studies hinders the assessment of the durability of treatment outcomes and the risk of late complications. Specifically, no follow-up data were available for the studies by Pathak et al and Sipos et al, further limiting the ability to evaluate postoperative outcomes over time.^19,20^ Finally, the doses of TXA varied across studies. Although TXA demonstrates superiority over placebo in reducing blood loss, further RCTs are necessary to evaluate the efficacy of local TXA in RM.

## CONCLUSIONS

This systematic review and meta-analysis of local TXA application in RM demonstrated notable advantages, particularly in reducing 24 h drain output, decreasing postoperative complications, and when used in higher concentrations, lowering the hematoma rates. The evidence also suggests that local TXA may help reduce the incidence of postoperative seromas, although this result did not achieve statistical significance. Nevertheless, local TXA did not have a significant impact on reducing the drain duration following RM. Although TXA showed promise, further RCTs are necessary to confirm its efficacy, determine the optimal dosage, and evaluate long-term outcomes.
